# Multisystemic Disease and Septicemia Caused by Presumptive *Burkholderia pseudomallei* in American Quarter Horse, Florida, USA

**DOI:** 10.3201/eid3107.241009

**Published:** 2025-07

**Authors:** Jason J. Thornton, John F. Roberts, David P. AuCoin, Apichai Tuanyok

**Affiliations:** University of Florida College of Veterinary Medicine, Gainesville, Florida, USA (J.J. Thornton, J.F. Roberts, A. Tuanyok); University of Nevada School of Medicine, Reno, Nevada, USA (D.P. AuCoin)

**Keywords:** melioidosis, bacteria, zoonoses, antimicrobial resistance, *Burkholderia pseudomallei*, *Equus caballus*, septicemia, multisystemic disease, Florida, USA

## Abstract

We report a presumptive case of melioidosis caused by an atypical *Burkholderia pseudomallei* serotype in an American quarter horse in north-central Florida, USA, through archived formalin-fixed paraffin-embedded specimens dating back to 2006. This case underscores the potential pathologic impact of emergent *B. pseudomallei* in the Gulf region of the United States.

*Burkholderia pseudomallei* is a gram-negative bacterium and the causative agent of the deadly disease melioidosis (*1*). This pathogen is a saprophytic bacillus distributed in the soil and water of tropical and subtropical environments. Regions where melioidosis is endemic include most of Southeast Asia, South America, the Caribbean, and northern Australia (*2*). Recently, *B. pseudomallei* was isolated from 3 unrelated patients from Mississippi, USA, who had no travel history to a melioidosis-endemic country (*3*–*5*). Those patients demonstrated symptoms consistent with melioidosis. Genetically similar organisms were isolated from the local soil and water, suggesting environmental transmission (*3*).

In addition to humans, many animal species, including horses, have been identified as susceptible to melioidosis (*6*,*7*). Clinical signs associated with melioidosis in animals mimic those of other virulent bacterial diseases and include lethargy, purulent nasal discharge, multiorgan abscesses, septicemia, and death by acute or chronic disease (*8*). Glanders, caused by the closely related *B. mallei*, can also cause similar clinical signs. *B. mallei* does not survive in the soil but can infect many species through animal–animal or zoonotic infection (*8*). Glanders has long been eradicated in the United States (*8*); however, this pathogen remains endemic in some regions of the Middle East, Asia, Africa, and Central and South America (*8*). Diagnosing either entity within the United States is critical because of the zoonotic potential of both organisms and the possible implications for public health. In addition, horses and livestock can be sentinel species for the environmental presence of *B. pseudomallei*, suggesting environmental contamination and posing risks to animals and humans. Predictive modeling studies indicate that *B. pseudomallei* might be ubiquitous throughout tropical and subtropical areas worldwide, including the southern United States (*4*,*5*).

The primary routes of *B. pseudomallei* infection are ingestion, inhalation, and percutaneous inoculation (*1*,*9*). The incidence of melioidosis increases dramatically after heavy rainfall (*9*). In addition, *B. pseudomallei* is classified as a category B bioterrorism bacterium and a Tier 1 (top tier) agent by the Centers for Disease Control and Prevention and Tier 1 by the US Department of Agriculture (*1*). Moreover, *B. pseudomallei* is highly resistant to antimicrobial drugs commonly used to treat sepsis in humans and animals, and an effective vaccine has not been approved (*1*). Furthermore, in apparently successfully treated humans and animals, relapses are common and precede development of chronic melioidosis (*9*). This article discusses a presumptive case of *B. pseudomallei* causing melioidosis-like diseases in an American quarter horse (*Equus caballus*) in Florida, USA. 

## The Study

**Figure 1:**
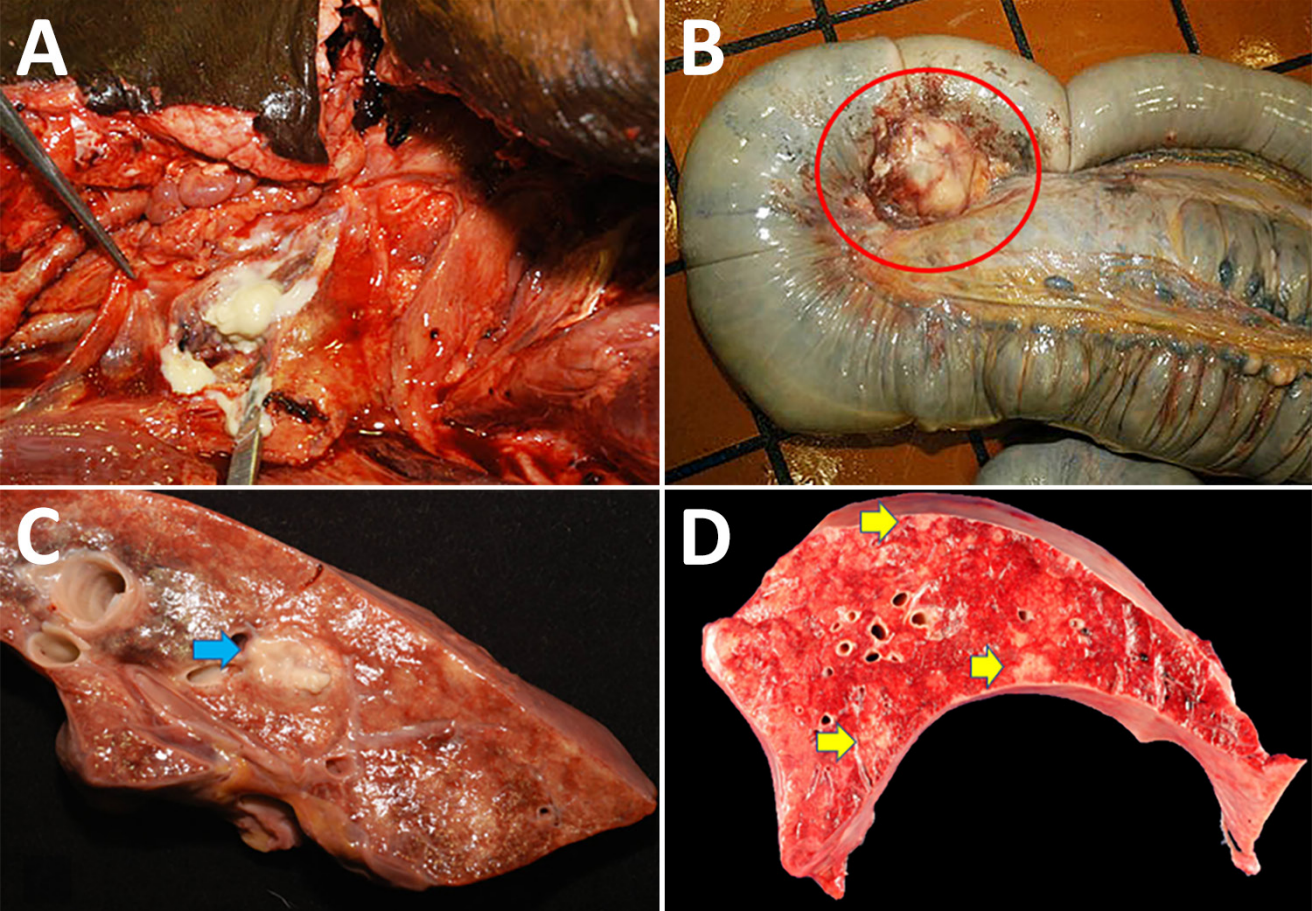
Postmortem photographs of an American quarter horse in study of multisystemic disease and septicemia caused by presumptive *Burkholderia pseudomallei*, Florida, USA. A) The submandibular lymph node was enlarged up to 20 cm in diameter and almost completely effaced by purulent material that extended into the retropharyngeal space and drains near the mandibular ramus. B) Cecal lymph nodes were diffusely enlarged up to 6 cm in diameter and filled with purulent material (red circle). C) The lung was diffusely firm with rib impressions and multifocal 5 × 5-mm to 1 × 2-cm abscesses (blue arrow). D) The liver contained multifocal random 1 × 1-cm pale tan foci scattered throughout the hepatic parenchyma (yellow arrows).

In 2006, an 8-year-old quarter horse gelding with a left retropharyngeal abscess was seen at the University of Florida Veterinary Medical Center (Gainesville, Florida, USA). Clinical examination and radiographs revealed a well-circumscribed 20 × 10 × 18-cm round soft tissue mass caudal to the ramus of the mandible with displacement of the left guttural pouch. Other examination findings included diffuse interstitial pneumonia, multiple cutaneous ulcers on the dorsal midline, anterior uveitis in the right eye, enlarged mesenteric lymph nodes, and an aneurysm of the right renal artery. Because of the animal’s declining clinical condition, it was humanely euthanized. At necropsy examination, gross findings included a large, soft, round abscess in the retropharyngeal space, compressing the guttural pouch with a draining tract into the epidermis (Figure 1, panel A). The mesenteric lymph nodes were diffusely enlarged, ranging from 2.0 to 6.0 cm in diameter, with purulent material and hemorrhage (Figure 1, panel B). Other gross findings included diffuse interstitial pneumonia with multifocal 2.0 × 2.0 × 2.0-cm peribronchiolar abscesses (Figure 1 panel C). Small 1.0 × 1.0 × 1.0-cm randomly scattered areas of necrosis were multifocally scattered in the liver (Figure 1, panel D); anterior uveitis was present in the right eye.

**Figure 2:**
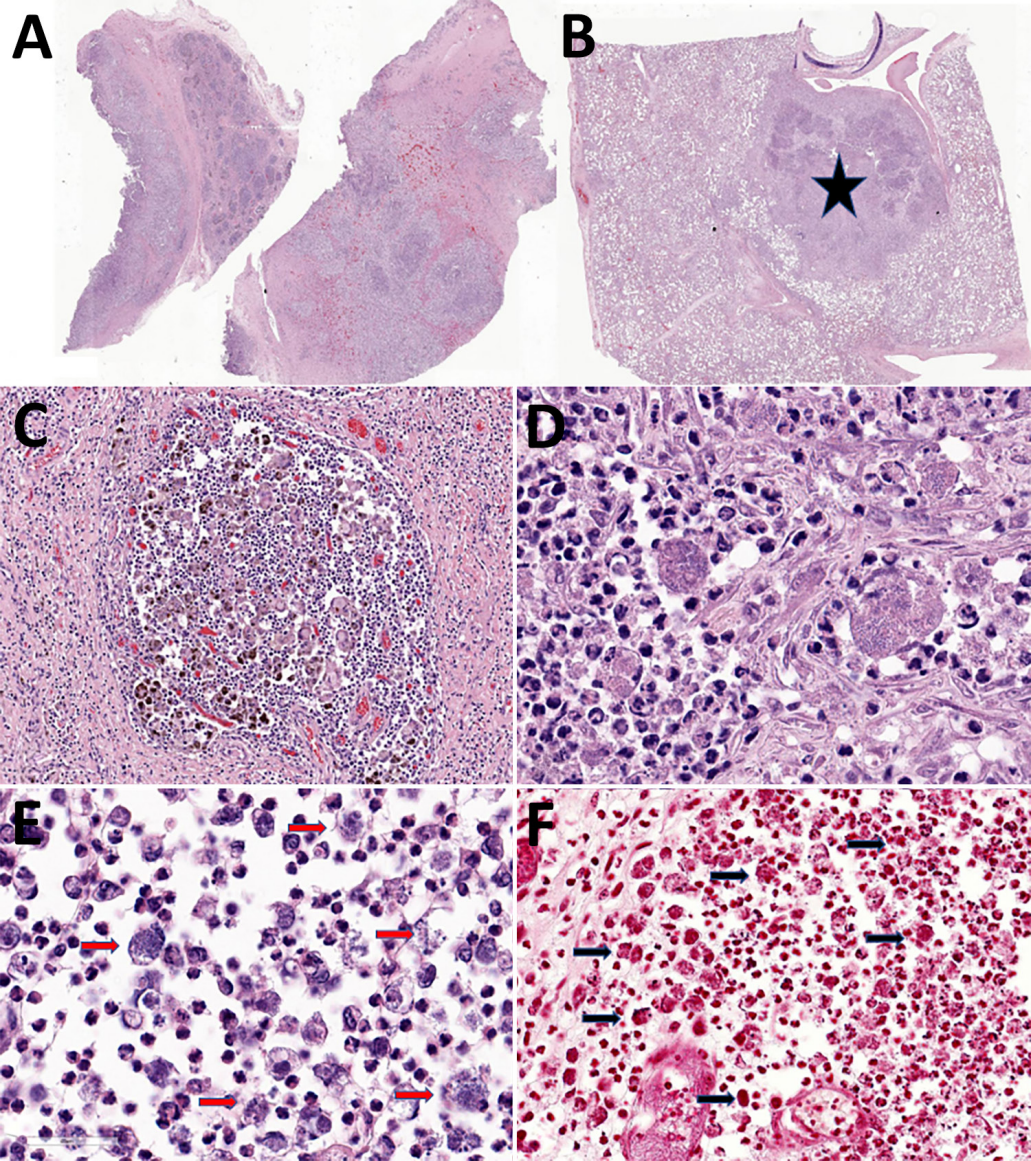
Photomicrography of tissues from study of multisystemic disease and septicemia caused by presumptive *Burkholderia pseudomallei* in American quarter horse, Florida, USA. Photomicrographs show hematoxylin and eosin staining of submandibular lymph node (LN) (A, C, E) and lung (B, D) and Gram stain of the submandibular LN (F). A) Submandibular LN shows multifocal to coalescing pyogranulomas. Original magnification ×2. B) The lung contains a parabronchial abscess (star). Original magnification ×2. C) The submandibular LN pyogranuloma contains necrotic debris, suppurative inflammation, and numerous hemosiderin laden macrophages, multinucleated giant cells surrounded by fibrosis. Original magnification ×20. D) Macrophages and neutrophils in the pulmonary abscess contain a mixed population of 1–2 μm coccobacilli. Original magnification ×40. E) The submandibular node neutrophils and macrophages contain numerous intracellular 1–2 μm bacilli (red arrows). Original magnification ×40. F) The bacilli in the submandibular lymph node are diffusely gram-negative (black arrows). Original magnification ×30.

Tissue specimens were fixed in 10% neutral buffered formalin, processed routinely, and embedded in paraffin. Paraffin-embedded sections were cut 4 μm thick and examined after staining with hematoxylin and eosin. We performed histochemical Gram stain to screen for bacteria. Histologic sections from the affected submandibular lymph node (Figure 2, panel A), lung (Figure 2, panel B), and mesentery revealed multifocal to coalescing abscesses and pyogranulomas containing necrotic cellular debris and degenerative neutrophils mixed with hemosiderophages (Figure 2, panels C–E). Numerous macrophages and neutrophils contained intracytoplasmic 1–2 µm gram-negative bacilli (Figure 2, panel F).

Postmortem aerobic culture of the retropharyngeal and mesenteric lymph nodes revealed pure *B. cepacia* complex growth. A fatty acid analysis using gas chromatography of the bacterial isolate supported this finding. *B. cepacia* complex is a heterogeneous group of bacteria from the *Burkholderia* genus that typically causes opportunistic infections in immunocompromised hosts. Disease outbreaks often occur in the hospital setting through contaminated medical devices or in patients with chronic respiratory diseases, such as cystic fibrosis (*10*).

The clinical manifestations of this case were unusual for *B. cepacia* complex and more consistent with the clinical course of acute, highly pathogenic *Burkholderia* species. We suspect that the organism was misidentified. The precise methodology of the original diagnosis was not reported in the case documents and is unknown to the authors of this report. Current biochemical systems in historically nonendemic areas often mistakenly identify *B. pseudomallei* strains as members of *B. cepacia* complex (*11*). Our team concluded that further investigation was necessary. Unfortunately, because of DNA fragmentation during routine tissue processing for histopathology, attempts at isolating bacterial DNA from the formalin-fixed paraffin-embedded tissue samples were unsuccessful. A limitation of this study is the inability to extract bacterial DNA from the tissue block, preventing definitive identification of *B. pseudomallei*.

**Figure 3:**
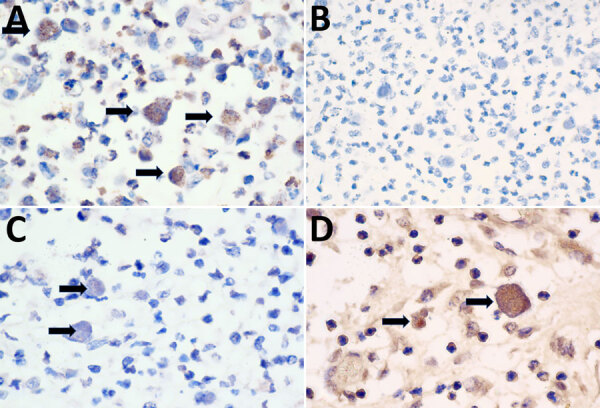
Immunohistochemical examination of tissues from American quarter horse with multisystemic disease and septicemia caused by presumptive *Burkholderia pseudomallei*, Florida, USA. Photomicrographs show immunohistochemistry for monoclonal antibodies 3A2 (A), 4C7 (B), 4C4 (C), and 5B4 (D) of the submandibular lymph node. A) Intra-phagocytic bacilli demonstrated strong immunoreactivity to 3A2 monoclonal antibody to *B. pseudomallei* lipopolysaccharides (LPS) O-Ag B/B2 (black arrows). Original magnification ×100. B) Intra-phagocytic bacilli were diffusely immunonegative for 4C7 monoclonal antibody to *B. pseudomallei* LPS O-ag serotype A. Original magnification ×40. C) Intra-phagocytic bacilli demonstrated weak multifocal immunoreactivity to 4C4 monoclonal antibody to *B. pseudomallei* capsular polysaccharides (black arrows). Original magnification ×100. D) Intra-phagocytic bacilli demonstrated strong immunoreactivity to 5B4 monoclonal antibody to *B. pseudomallei* LPS O-Ag B/B2 (black arrows). Original magnification ×100. Bacilli were immunonegative to 3D11 (not shown) monoclonal antibody to *Bm* LPS O-Ag.

*B. pseudomallei* employs a network of polysaccharides, including capsular polysaccharides (CPS) and lipopolysaccharides (LPS), to enhance virulence and immune evasion (*12*,*13*). A panel of monoclonal antibodies targeting *B. pseudomallei* and *B. mallei* CPS (4C4), typical LPS O-Ag serotype A (4C7), atypical LPS O-Ag serotype B and its variant B2 (3A2, 5B4), and *B. mallei* LPS O-Ag (3D11) were used (*13*). The secondary antibody was biotinylated goat anti-mouse IgG (1:200 dilution). Intrahistiocytic bacilli were immunopositive for 4C4, 3A2, and 5B4 and immunonegative for 4C7 and 3D11 (Figure 3). We tested 19 different strains of *B. cepacia* complex and demonstrated no cross-reactivity to 4C4, 4C7, 3A2, and 5B4 on Western blot analysis (Table). Previous studies have shown *B. pseudomallei* cross-reactivity by some *B. cepacia* complex strains to *B. pseudomallei*–like CPS-specific antibodies (4C4) (*14*) and *B. mallei* to monoclonal antibodies to typical type A LPS (4C7); however, cross-reactivity of either species to *B. pseudomallei* atypical type B LPS (3A2 and 5B4) has not been reported, and cross-reactivity was not noted in our experiments (*15*). The immunohistochemistry and Western blot results suggest infection with *B. pseudomallei* with atypical O-Ag type B or B2.

**Table:** Monoclonal antibody labeling through immunohistochemistry or Western blot analysis of various strains of *Burkholderia* species in study of multisystemic disease and septicemia caused by presumptive *Burkholderia pseudomallei* in American quarter horse, Florida, USA

Monoclonal antibody	Florida case *B. pseudomallei*	*B. pseudomallei* MSHR840, serotype B2	*B. pseudomallei* Bp82, serotype A	*B. pseudomallei* 576mn, serotype B	*B. cepacia* complex (*12*), n = 19	*B. mallei* ATCC 23344, serotype A variant
4C4, Bp CPS	+	+	+	+	–	–
4C7, Bp O-Ag serotype A	–	–	+	–	–	–
3A2, Bp O-Ag serotype B	+	+	–	+	–	–
5B4, Bp O-Ag serotype B2	+	+	–	+	–	–
3D11, Bm O-Ag serotype A variant	–	–	–	–	–	+

## Conclusions

This case report describes the histomorphology and immunohistochemical identification of an atypical serotype of *B. pseudomallei* in a horse with clinical signs consistent with melioidosis. Although type A is the most common LPS O-Ag type of *B. pseudomallei*, accounting for most infections, types B and B2 are found more frequently in Australia (*13*). The route of exposure and travel history of this horse is unknown. On the basis of the immunohistochemistry results in this case, we conclude that the initial culture and biochemical analysis misidentified *B. cepacia* complex. Western blot assay analysis of purified LPS from numerous bacteria in the *B. cepacia* complex failed to highlight any of the mentioned monoclonal antibodies. This case underscores the potential pathological effects of *B. pseudomallei* in horses and other animals in the United States, emphasizing the need for increased awareness and understanding of its emergence as a potential pathogen in diverse species.
